# Genetic risk assessment of lethal prostate cancer using polygenic risk score and hereditary cancer susceptibility genes

**DOI:** 10.1186/s12967-023-04316-y

**Published:** 2023-07-06

**Authors:** Xiaohao Ruan, Da Huang, Jingyi Huang, James Hok-Leung Tsu, Rong Na

**Affiliations:** 1 Department of Urology, Ruijin Hospital, Shanghai Jiao Tong University School of Medicine, Shanghai, 200025 China; 2 Division of Urology, Department of Surgery, Queen Mary Hospital, The University of Hong Kong, Hong Kong, China

**Keywords:** Benign prostatic hyperplasia, Germline mutation, Polygenic risk score, Prostate cancer

## Abstract

**Background:**

The genetic risk of aggressive prostate cancer (PCa) is hard to be assessed due to the lack of aggressiveness-related single-nucleotide polymorphisms (SNPs). Prostate volume (PV) is a potential well-established risk factor for aggressive PCa, we hypothesize that polygenic risk score (PRS) based on benign prostate hyperplasia (BPH) or PV-related SNPs may also predict the risk of aggressive PCa or PCa death.

**Methods:**

We evaluated a PRS using 21 BPH/PV-associated SNPs, two established PCa risk-related PRS and 10 guideline-recommended hereditary cancer risk genes in the population-based UK Biobank cohort (N = 209,502).

**Results:**

The BPH/PV PRS was significantly inversely associated with the incidence of lethal PCa as well as the natural progress in PCa patients (hazard ratio, HR = 0.92, 95% confidence interval [CI]: 0.87–0.98, *P* = 0.02; HR = 0.92, 95% CI 0.86–0.98,* P* = 0.01). Compared with men at the top 25th PRS, PCa patients with bottom 25^th^ PRS would have a 1.41-fold (HR, 95% CI 1.16–1.69, *P* = 0.001) increased PCa fatal risk and shorter survival time at 0.37 yr (95% CI 0.14–0.61, *P* = 0.002). In addition, patients with *BRCA2* or *PALB2* pathogenic mutations would also have a high risk of PCa death (HR = 3.90, 95% CI 2.34–6.51, *P* = 1.79 × 10^–7^; HR = 4.29, 95% CI 1.36–13.50, *P* = 0.01, respectively). However, no interactive but independent effects were detected between this PRS and pathogenic mutations.

**Conclusions:**

Our findings provide a new measurement of PCa patients’ natural disease outcomes via genetic risk ways.

**Supplementary Information:**

The online version contains supplementary material available at 10.1186/s12967-023-04316-y.

## Introduction

Prostate cancer (PCa) is one of the most common and lethal cancers worldwide. It ranked 3rd in new cancer cases and 8th in cancer deaths with approximately 1.4 million new cases and 37,500 deaths in 2020 [1]. The common use of prostate-specific antigen (PSA) screening has contributed to the early detection of PCa and the increased number of PCa incidences in statistics. However, it might not be able to distinguish clinically aggressive cancer from low-risk cases and has relatively low socioeconomic efficacy without selectively being applied [2]. Early detection is critical for high-risk individuals to receive effective intervention at the disease’s early stage, especially for men with an increased risk of clinically significant PCa (advanced or lethal PCa).

Genetic risk assessment is one of the common tools to identify individuals at risk to develop diseases. It usually contains three components: family history (FH), inherited pathogenic/likely pathogenic (P/LP) variants in cancer susceptibility genes (such as *BRCA2*, *HOXB13*), and single nucleotide polymorphism (SNP)-based polygenic risk scores (PRS). The relationship between genetic risk and aggressive PCa is still under exploration. About ~ 3% of the general population was observed with a positive FH but it was not associated with clinically significant PCa [3]. In addition, less than 2% of men in the population and ~ 5% of PCa patients would carry a P/LP variant [4]. P/LP variants in *BRCA1/2*, *ATM,* and *CHEK2* (c.1100 delC) were reported to be associated with PCa aggressiveness [5–9]. The National Comprehensive Cancer Network (NCCN) guideline also recommended basic germline testing in hereditary cancer susceptibility genes if patients had a PCa FH, Ashkenazi Jewish ancestry, a personal history of certain cancers and aggressive PCa [10]. However, the cancer-specific lethal risk of these P/LP variants of cancer susceptibility genes was rarely reported in incidental PCa patients. PCa-risk-associated SNPs-based PRS could identify additional individuals at high PCa risk and the clinically relevant endpoint of PCa death with a wide range at a population level [6, 11]. But it fails to differentiate indolent PCa and advanced PCa [7]. To date, it is difficult to assess the genetic risk of aggressive PCa or disease outcomes due to the lack of aggressive-associated SNPs. Only eight mixed-ethnical small-scale GWAS reported 16 SNPs associated with aggressive PCa (ten independent SNPs reached the genome-wide significant level and four SNPs had been validated) [12]. And the standard in the definition of aggressive PCa and the validation were divided across these GWASs.

Established evidence shows that a low prostate volume (PV) is significantly associated with clinically significant PCa [13–15]. However, the change of PV or benign prostatic hyperplasia (BPH) happens most likely in men’s 70 s [16]. To evaluate the possibility of an enlarged PV or BPH diagnosis at a younger age, we would use a PRS as an instrument to assess the genetic risk of BPH/PV. The present study hypothesizes that BPH/PV PRS may predict individuals’ risk of BPH/PV as well as the risk of PCa death. We will evaluate whether it could predict PCa death and provide additional assessment over P/LP variants of hereditary cancer susceptibility genes (*ATM*, *BRCA1*, *BRCA2*, *CHEK2*, *PALB2*, *MLH1*, *MSH2*, *MSH6*, *PMS2*, and *HOXB13*).

## Materials and methods

### Study population

**Fig. 1:**
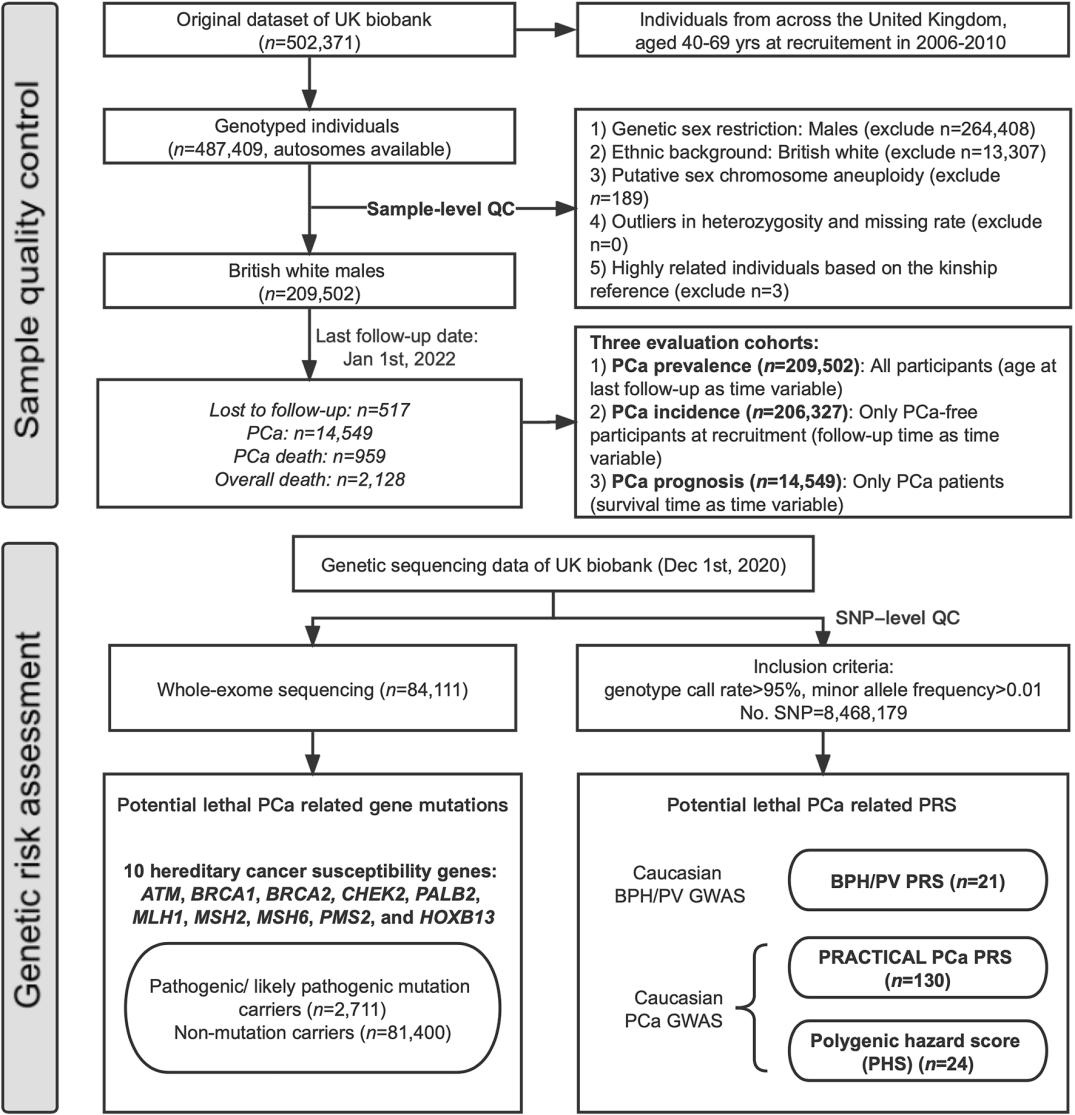
Workflow for genetic risk evaluation and comparison

The current study was conducted in a large cohort from the UK Biobank (UKB) [17]. Only British white males were included in this study. PCa diagnosis (ICD10: C61) was based on records from national cancer registries and self‐reports. Lethal PCa is identified based on records of the primary cause of death from national death registries. PCa as the contributory (secondary) cause of death was therefore considered as an accompanied condition. Self‐reported PCa family history (FH) in fathers or brothers, ethnic background, age at recruitment, age at death, BPH diagnosis (ICD10: N40) were also obtained. Charlson Comorbidity Index score was calculated based on the presence of 17 conditions, each of which is assigned a weighted score of 1, 2, 3, or 6 [18]. All participants provided written informed consent to take part in the study. The study was approved by the Northwest Multi-centre Research Ethics in Manchester, UK (IRAS project ID: 299,116; Application No. 66813). Three different evaluation cohorts were presented in Fig. 1, and the used data-field ID for each phenotype in UKB was in Additional file 1: Table S1.

### Genotyping and whole exome sequencing

Approximately 97% and 40% of UKB subjects had genotyping data and whole exome sequence (WES) data, respectively, provided by the UKB (October 20, 2020). The UKB samples were genotyped via the UK Biobank Axiom array and UK BiLEVE array [19]. Participants were excluded if: (1) they were not genetic males; (2) their sex chromosome karyotypes were putatively different from XY; (3) they were outliers in heterozygosity and missing rates; and (4) they were highly related individuals based on the kinship reference [20]. Genotyping data was provided with performed imputation and quality control [19]. SNPs were excluded if they had: (1) genotype call rate < 95%; (2) minor allele frequency < 0.01. PLINK 2.0 software was used for calculating 10 principal components (PCs) after linkage disequilibrium pruning [21]. Whole exome sequencing data in VCF form was also obtained. Pathogenic/likely pathogenic (P/LP) mutations in the 10 hereditary cancer susceptibility genes (*ATM*, *BRCA1*, *BRCA2*, *CHEK2*, *HOXB13*, *PALB2*, *MLH1*, *MSH2*, *MSH6* and *PMS2*) were annotated following the general sequence variant interpretation guidelines of the ACMG [22]. Details of sample quality control was described in Fig. 1.

### PRS calculation

Independent genome-wide significant risk variants were selected (*n* = 27 for BPH [23, 24], Additional file 1: Table S2) from the evidence-based published GWAS in the Icelandic BPH/LUTs dataset [23] (*n* = 113,443) and the Electronic Medical Records and Genomics Network (eMERGE) [24] (*n* = 6,672). We only used the effective sizes of the independent SNPs from these two cohorts for constructing the BPH/PV PRS, without including the results from UKB. We used PRSice2 software to calculate PRS with GWAS clumped results (range 250 kb, r^2^ = 0.1) and summary association statistics [25]. A weighted PRS for each patient was calculated by summing the number of risk alleles at each of the 27 SNPs multiplied by the logarithm of the SNP’s Odds ratio. The previously reported lethal PCa risk-related PRACTICAL PRS (No. SNPs = 147) [11, 26] and Polygenic hazard score (PHS, No. SNPs = 46) [27] were also calculated in this way (Additional file 1: Table S3, S4). Details of the evaluation was described in Fig. 1.

### Statistical analyses

Baseline characteristics were illustrated by descriptive statistics. Chi-squared test was used to compare the difference between categorical variables. Student’s *t*-test was applied to evaluate the normally distributed continuous variables while the Mann–Whitney U test was used to evaluate non-normally distributed continuous variables and the trend for the ranks across ordered groups. Crude PCa-specific mortality was calculated from the date of the first diagnosis of PCa to the date of death or the last date of follow-up updated on Jan 1st, 2022. Univariable or multivariable analyses were performed to test for the independent effect of factors associated with PCa death using logistic and Cox proportional hazards regression models (in participants with no kinship found). The “Coxme” R package was used for building multi-level mixed effects cox models for genetic relatedness clustered within participants [28]. Survival time after PCa diagnosis and the differences across subgroups were estimated with restricted mean survival time (RMST) [29]. The multiplicative interaction was quantified by including a product term of BPH/PV genetic risk and the presence of germline P/LP mutation events in the model. The additive interaction was measured by calculating the relative excess risk due to interaction and the attributable proportion due to the interaction based on coefficients of the product term with R package “interaction” [30].

All Statistical analyses were performed using R version 4.1.2 [31] and a 2-tailed *p* < 0.05 was considered statistically significant.

## Results

The demographic characteristics and the baseline information of the prospective cohort were shown in Table 1**,** while the prevalence data were in Additional file 1: Table S5. During a mean follow-up time of 8.0 yr, a total of 14,549 British white males were diagnosed with PCa, of which 959 (6.6%) died of PCa, 1169 (8.0%) died due to other reasons, 7 (0.05%) lost to follow-up and 12,414 (85.3%) survived till the latest follow-up in Jan 1st, 2022. Men who died of PCa were less likely to have a BPH diagnosis (15.7% vs. 19.6%, *P* = 0.004) compared to men with non-lethal disease. PCa patients without a BPH history would have a higher PCa-specific mortality rate (crude PCa-specific mortality rate in males without BPH was 8.49 per 1000 men vs. 6.91 per 1000 men in males with BPH, rate ratio, RR = 1.28, 95% confidence interval, 95% CI 1.08–1.56, *P* = 0.004). There was no difference in the family history of PCa (*P* > 0.9) between the two groups (Table 1). Besides the increasing age, a higher score on the Charlson Comorbidity Index (indicating a heavier burden of illness) was also associated with an increased lethal PCa risk (*P* < 0.001, Table 1).

**Table 1:** Demographic and clinical characteristics of prostate cancer patients in UK Biobank

Characteristics	Lethal PCa	Non-lethal PCa	All	P value^b^
Male participants, No. (%)	959 (6.6)	13,590 (93.4)	14,549	
Age at diagnostic, mean (SD), yr	65.8 (6.2)	66.9 (6.5)	66.9 (6.5)	0.013
Age at death, mean (SD), yr	71.6 (5.3)	73.5 (5.1)^a^	72.7 (5.3)	< 0.001
BPH diagnosis, No. (%)				0.004
Yes	151 (15.7)	2657 (19.6)	2808 (19.3)	
No	808 (84.3)	10,933 (80.4)	11,741 (80.7)	
Family history of PCa, No. (%)				0.949
Yes	114 (11.9)	1617 (11.9)	1731 (11.9)	
No	799 (83.3)	11,402 (83.9)	12,201 (83.9)	
Missing	46 (4.8)	571 (4.2)	617 (4.2)	
Charlson comorbidity index scores				< 0.001
0	108 (11.3)	4441 (32.7)	4549 (31.3)	
1–2	112 (11.7)	5906 (43.4)	6018 (41.4)	
> 2	739 (77.0)	3243 (23.9)	3982 (27.3)	
Genetic kinship				0.098
No kinship found	644 (67.2)	9447 (69.5)	10,091 (69.3)	
At least one relative identified	314 (32.7)	4136 (30.4)	4450 (30.6)	
Ten or more third-degree relatives	1 (0.1)	7 (0.1)	8 (0.1)	
WES data, No. (%)	392 (40.9)	5504 (40.5)	5896 (40.5)	0.808

PCa, Prostate cancer; BPH, Benign prostate hyperplasia; N/A, Not applicable; WES, Whole-exome sequencing

^a^1173 deaths due to other reasons, not for PCa as primary death cause

^b^Chi-squared test or t test was used to compare lethal PCa group and Non-lethal PCa group

A total of 21 SNPs were used to calculate BPH/PV PRS finally (Additional file 1: Table S2). And the Odds ratios (ORs) per 1-SD increment in BPH-related PRS represented 1.75 (OR, 95% CI 1.68–1.83) increased risk to have BPH (*P* < 0.001). Due to mismatched allele codes, we only matched 130 out of 147 SNPs and 24 out of 47 SNPs reported in the PRACTICAL PRS [11, 26] and PHS [27](established lethal PCa-related PRS), respectively (Additional file 1: Table S3, S4).

**Fig. 2:**
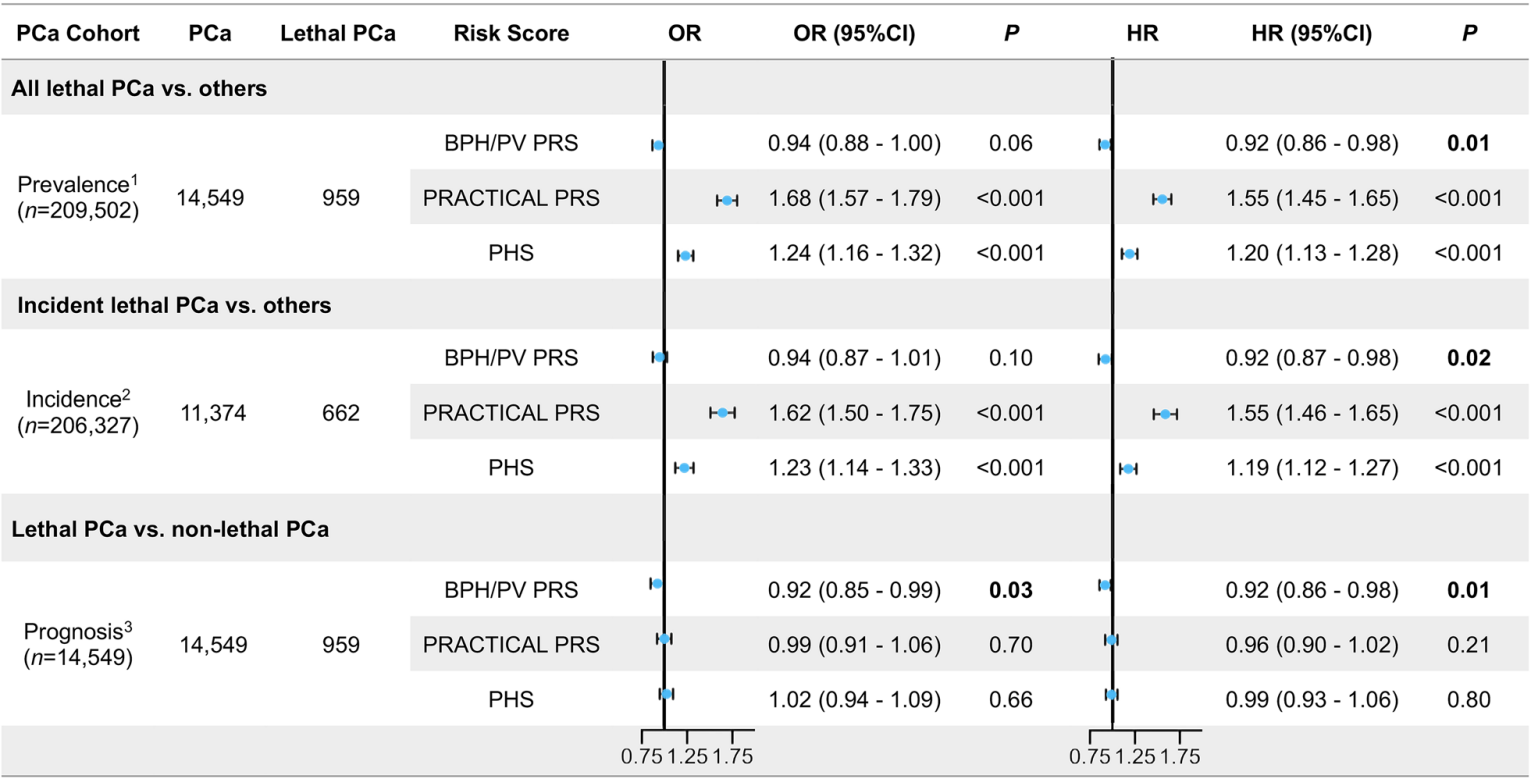
Multivariate analysis of associations between lethal prostate cancer among BPH/PV risk score or established prostate cancer risk scores. PCa, Prostate cancer; OR, Odds ratio; HR, Hazard ratio; CI, Confidence interval; PHS, Polygenic hazard score; BPH, Benign prostate hyperplasia; PV, prostate volume; SNP, Single nucleotide polymorphism; PRS, Polygenic risk score. The risk scores were standardized within each cohort by dividing by standard deviation. **1** PCa prevalence cohort: the cohort included all patients and used patient’s age at death or at last follow-up (2022 Jan 1st or lost) as time variable for mixed-effect Cox regression. Multivariate ORs were adjusted for age at last follow-up, family history, Charlson Comorbidity Index score, genotyping chip batches and 10 principal components. Multivariate HRs were adjusted for the same factors except for age. **2** PCa incidence cohort: the cohort excluded PCa patients before recruitment and used follow-up time as time variable for mixed-effect Cox regression. Multivariate ORs or HRs were adjusted for age at recruitment, family history, Charlson Comorbidity Index score, genotyping chip batches and 10 principal components. **3** PCa prognosis cohort: the cohort excluded patients without PCa at last follow-up and used survival time as time variable for mixed-effect Cox regression. Multivariate ORs or HRs were adjusted for age at onset, family history, Charlson Comorbidity Index score, genotyping chip batches and 10 principal components

The comparison between BPH/PV PRS and reported PCa PRSs (PRACTICAL PRS and PHS) was presented in Fig. 2. BPH/PV PRS was not only significantly associated with PCa-specific mortality (Hazard ratio, HR = 0.92, 95% CI 0.86–0.98, *P* = 0.01) in PCa patients, but also associated with incident PCa death (HR = 0.92, 95% CI 0.86–0.98, *P* = 0.01; HR = 0.92, 95% CI 0.87–0.98, *P* = 0.02) at a population level in different time-set. The association remained significant after excluding participants with at least one relative identified (Additional file 1: Table S6). The PRACTICAL PRS and the PHS performed similarly well in predicting lethal PCa to those previously reported, especially in the prevalence and incidence cohort (both *P* < 0.05). However, they showed no significant relation with lethal PCa outcomes in PCa patients (*P* > 0.05). The survival analysis in the entire PCa cohort revealed that men with lower BPH/PV PRS might have a significantly shorter survival time (Log-rank test *P* = 0.004). Compared with those having top BPH/PV PRS (75–100th percentile, the highest risk of BPH), PCa patients with lowest BPH/PV PRS (0–25th percentile, the lowest risk of BPH) would have a 1.41-fold increased risk of PCa death during the follow-up (HR, 95% CI 1.17–1.71, *P* = 0.001, Table 2), as well as a relatively lower long-term survival probability at 83.7% (20-year survival probability, 95% CI 80.4–87.1%, *P* for trend < 0.001) and shorter survival time of 0.37 yr (RMST, 95% CI 0.14–0.61, *P* = 0.002).

**Table 2:** Associations between categorized BPH/PV-related PRS and risk of prostate cancer-specific mortality in prostate cancer prognosis cohort

	75–100th PRS	50–75th PRS	25–50th PRS	0–25th PRS	*P-trend*
Mean age at PCa death (SD), yr	72.2 (5.0)	71.9 (5.1)	71.6 (5.7)	71.1 (5.5)	0.17
Proportion of deceased	5.5% (199/3,638)	6.6% (241/3637)	7.1% (260/3,637)	7.1% (259/3,637)	0.01
OR^a^ (95%CI)	1 (Ref.)	1.36 (1.10–1.69)	1.44 (1.16–1.79)	1.37 (1.10–1.70)	4.95 × 10^–3^
HR^a^ (95%CI)	1 (Ref.)	1.33 (1.11–1.61)	1.34 (1.11–1.62)	1.41 (1.17–1.71)	7.52 × 10^–4^
5-year survival probability (95% CI, %)	96.3 (95.7–97.0)	95.9 (95.2–96.6)	94.9 (94.1–95.6)	94.8 (94.0–95.6)	3.10 × 10^–3^
10-year survival probability (95% CI, %)	93.5 (92.6–94.5)	92.2 (91.2–93.3)	91.7 (90.6–92.8)	91.6 (90.3–92.6)	8.44 × 10^**–**5^
20-year survival probability (95% CI, %)	87.4 (84.0–90.8)	84.5 (81.4–87.8)	84.2 (81.0–87.5)	83.7 (80.4–87.1)	< 2.20 × 10^**–**16^
Restricted mean survival time (95% CI, %)	0 (Ref.)	− 0.01 (− 0.26 to 0.24)	− 0.09 (− 0.34 to 0.15)	− 0.37 (− 0.61 to − 0.14)	1.88 × 10^–3^

PCa, Prostate cancer; vs., versus; OR, odds ratio; CI, confidence interval; Ref, reference

^a^OR and HR is calculated by logistic and cox mixed-effect model (based on sample relatedness) adjusted for kinship, age at onset, charlson comorbidity index score, genotyping batches, and 10 principal components

**Fig. 3:**
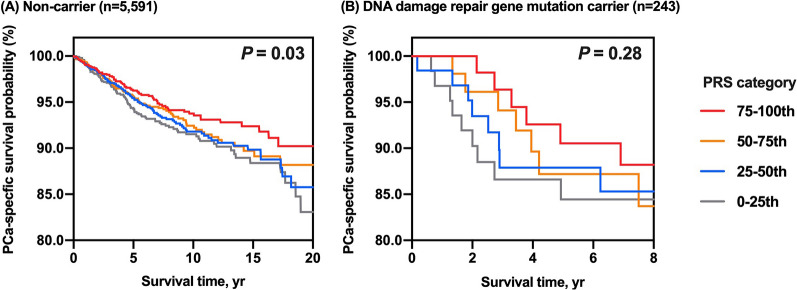
Kaplan–Meier survival curves for mutation non-carriers and carriers across BPH/PV PRS categories. **A** in the non-carriers of PCa patients (n = 5,591, log-rank test for trend, *P* = 0.03), **B** in the DNA damage repair genes carriers (*n* = 243, log-rank test for trend, *P* = 0.28). PCa, prostate cancer; DDR, DNA damage repair

The carrier rates of P/LP mutation in the UKB unselected PCa patients (data available in 5896 PCa patients) for each of the ten genes were shown in Table 3, ranging from 0.02% for *MLH1* to 1.64% for *CHEK2* (Mutations listed in Additional file 1: Table S7). *BRCA2* and *PALB2* were significantly associated with PCa death, with HR estimated at 3.90 (95% CI 2.34–6.51, *P* < 0.001) and 4.31 (95% CI 1.36–13.50, *P* = 0.01). The estimated RMST up to the minimum of the largest observed event time within the group (14.38 yrs) is 10.34 yrs vs. 13.57 yrs for *BRCA2* + group vs. *BRCA2*- group and the survival time difference is 3.23 yrs (95% CI 1.55–4.90, *P* < 0.001). However, only the *BRCA2* + vs. *BRCA2*- group was different in terms of RMST. This means that only *BRCA2* mutation status rather than mutation status in other genes are significantly associated with shorter survival time after PCa diagnosis. To be more specific, mutations in the other eight genes indicated by the NCCN guideline were not significantly associated with survival time in this cohort (*P* > 0.05). When investigating the interaction between mutation status and BPH/PV PRS, additional analyses were performed in subgroups of patients based on the carrier status (Fig. 3). In non-carriers, a decreased BPH/PV PRS was significantly associated with an increased risk of PCa death (Log-rank *P* = 0.03, Fig. 3A). However, the trend was not observed among DNA damage repair (DDR) gene carriers (Log-rank *P* = 0.28, Fig. 3B). And no significant multiplicative or additive effects between BPH/PV PRS and hereditary gene mutations (*BRCA2* and *PALB2*) were detected (*P*_*multiplicative*_ = 0.65, P_*additive*_ = 0.71), as well as with DDR gene carriers (*P*_*multiplicative*_ = 0.88, P_*additive*_ = 0.66).

**Table 3:** Association of germline pathogenic mutations in guideline-recommended genes with lethal prostate cancer risk in the UK Biobank PCa patients with WES results

**Genes**	No. (%) of carrier			Cancer-specific mortality	
	Lethal PCa (n = 392)	Non-lethal PCa (n = 5504)	PCa patients (N = 5896)	HR^a^ (95% CI)	*P*
*BRCA2*	16 (4.1)	31 (0.56)	47 (0.80)	3.90 (2.34–6.51)	1.79 × 10^–7^
*PALB2*	3 (0.77)	6 (0.11)	9 (0.15)	4.29 (1.36–13.50)	0.01
*BRCA1*	1 (0.26)	9 (0.18)	10 (0.17)	1.93 (0.27–13.93)	0.51
*ATM*	3 (0.77)	43 (0.78)	46 (0.78)	1.13 (0.36–3.53)	0.83
*CHEK2*	8 (2.0)	89 (1.62)	97 (1.64)	1.35 (0.67–2.73)	0.40
*HOXB13*	5 (1.3)	63 (1.14)	68 (1.15)	1.16 (0.48–2.81)	0.74
*MSH2*	0	0	0	N/A	N/A
*MSH6*	2 (0.51)	22 (0.40)	24 (0.41)	1.50 (0.37–6.05)	0.57
*MLH1*	1 (0.26)	0	1 (0.02)	N/A	N/A
*PMS2*	1 (0.26)	11 (0.20)	12 (0.20)	1.08 (0.15–7.63)	0.95
*MMR (4 genes)*	4 (1.0)	33 (0.60)	37 (0.63)	1.65 (0.61–4.41)	0.33
*DDR (9 genes)*	35 (8.93)	208 (3.78)	243 (4.12)	2.13 (1.48–3.06)	5.12 × 10^–5^
*BRCA2/PALB2*	19 (4.8)	37 (0.67)	56 (0.95)	4.00 (2.50–6.41)	7.47 × 10^–9^

WES, Whole-exome sequencing; OR, Odds ratio; CI, Confidence interval; MMR, Mismatch repair genes (include *MSH2, MSH6, MLH1* and *PMS2*); DDR, DNA damage repair genes (include *BRCA1/2, ATM, CHEK2, MSH2, MSH6, MLH1, PMS2, PALB2*); N/A, not applicable

^a^Association results (cox mixed-effect model) were adjusted for kinship, age at diagnosis, Charlson Comorbidity Index score, batches and 10 principal components

## Discussion

In the present study, we derived a PRS based on the 21 reported independent risk variants for BPH in European ancestry, which showed an additional and considerable prognostic value for PCa. We also evaluated 10 guideline‐recommended familial cancer risk genes for their association with PCa fatality in the prospective PCa cohort. We found that: (1) BPH/PV PRS was significantly and independently associated with PCa death; (2) known PCa-related PRS (PRACTICAL PRS and PHS) could distinguish individuals’ PCa risk but failed to predict PCa patients’ natural outcome; and (3) Only the P/LP mutations in *BRCA2* or *PALB2* were found to be associated with PCa death in this cohort and did not interact with BPH/PV PRS.

As a well-established risk factor, the association between PV or BPH and PCa has been comprehensively investigated in many studies. Orsted et al.’s nationwide cohort study of 3,009,258 men showed males with BPH were more likely to develop PCa and die of it [32]. However, increased examination (PSA testing, digital rectal examination, and biopsies) in patients with BPH could lead directly to PCa detection as well as the consequence of PCa-related death. Other studies conducted that a relatively small PV would be associated with poorer PCa prognosis from prostatectomy or biopsy cohort. For instance, a series of studies on radical prostatectomy specimens showed that tumors arising in larger prostates tend to have favorable pathological features and biochemical progression [13–15]. Moreover, Lorenzo et al. suggested that the mechanical stress induced by BPH might impede prostatic tumor growth [33]. Thus, a measure of PV or BPH status could be a potential index for PCa aggressiveness. However, the change of PV or BPH happens most likely in men’s 70 s [15]. To evaluate the possibility of a changed PV or BPH diagnosis at a younger age, it would be useful to develop an instrument to provide an indirect measurement in advance. We, hence, focused on SNPs-based PRS which could provide consistent lifetime estimates for PV and BPH.

Many studies reported PRS using PCa risk-associated SNPs to evaluate PCa predisposition. Those PRSs were mainly designed for identifying individuals who may be predisposed to PCa rather than the likelihood of more aggressive or lethal PCa. PRS for PCa risk was not associated with Gleason Score, pathological stage, and biochemical recurrence [34]. Thus, it showed the same detection bias as family history or BPH in epidemiological research, ignoring the fact that increased discoveries of PCa could bring an elevated number of clinically significant PCa (Fig. 2). On the other hand, limited aggressive PCa-associated SNPs made it hard to establish a direct SNPs-based PRS for PCa prognosis. Therefore, with a logically sound hypothesis, sufficient clinical evidence, and underlined biological mechanisms, we proved that the BPH/PV PRS could be a tool for predicting aggressive PCa beyond other reported PRSs. However, the cumulative effect of BPH/PV PRS could be too mild to emerge under the relatively few DNA damage repair gene carriers.

Important strengths of this study include the prospective design and the relatively large sample size of the cases from a Caucasian population which increases the generalizability of our findings. Meanwhile, several limitations should also be noted. Firstly, the statistical power was reduced due to the limited number of available SNPs. If possible, we would repeat and enrich it in another validation cohort in the future. Due to the firm relation to PCa and considerable numbers of SNPs, the mismatched SNPs in PRACTICAL PRS and PHS only minimally affected the power of analysis and comparison results. Secondly, the relatively few P/LP mutation carriers affected the subgroup analysis, but a similar trend still existed. Further investigation would be worthy in a carrier-only cohort. Thirdly, the identification of aggressive PCa and indolent PCa was restricted by the nature of the UK Biobank database. We were unable to identify patients with aggressive phenotypes other than death, such as high Gleason Score, metastatic disease, etc. These phenotypes were equally important in terms of disease management and clinical intervention. Additionally, selection and information biases were inevitable: the self-selection among study participants and single ancestral makeup (overwhelmingly white and from northern European); some of the information collected in the study relied on self-reporting. However, the current study focusing on PCa death could also provide reliable evidence which might raise future interest in the evaluation of different disease outcomes.

In conclusion, BPH/PV PRS was significantly associated with PCa death and functioned as a potential prognostic assessment tool for PCa. Moreover, a combination of BPH/PV PRS and *BRCA2/PALB2* mutation status may help to identify high lethal PCa genetic risk and shorter survival time.

## Supplementary Information

**Additional file 1:** **Table S1.** The UKB data-field ID for each phenotype used in this study. **Table S2.** Benign prostatic hyperplasia association results for all 27 reported genome-wide significant risk variants. **Table S3.** Details on SNP information of PRACTICAL PRS. **Table S4.** Details on SNP information of PHS. **Table S5.** Demographic and clinical characteristics of British white males in UK Biobank. **Table S6.** Associations between BPH/PV-related PRS and risk of prostate cancer-specific mortality in participants with no kinship found. **Table S7.** Detail on Pathogenic/Likely Pathogenic Germline Mutation.

## Data Availability

Data used in this research are publicly available to qualified researchers on application to the UK Biobank (www.ukbiobank.ac.uk).

## Abbreviations

PCa: Prostate cancer
PRS: Polygenic risk score
BPH: Benign prostatic hyperplasia
PV: Prostate volume
PSA: Prostate-specific antigen
FH: Family history
SNP: Single nucleotide polymorphism
RMST: Restricted mean survival time
UKB: UK Biobank
