# Supplementary material for: Genetic risk assessment of lethal prostate cancer using polygenic risk score and hereditary cancer susceptibility genes
Source: J Transl Med. 2023 Jul 6;21:446. doi: 10.1186/s12967-023-04316-y (PMC10327136; doi:10.1186/s12967-023-04316-y)
Supplement: Supplementary file 1 — Additional file 1: Table S1. The UKB data-field ID for each phenotype used in this study. Table S2. Benign prostatic hyperplasia association results for all 27 reported genome-wide significant risk variants. Table S3. Details on SNP information of PRACTICAL PRS. Table S4. Details on SNP information of PHS. Table S5. Demographic and clinical characteristics of British white males in UK Biobank. Table S6. Associations between BPH/PV-related PRS and risk of prostate cancer-specific mortality in participants with no kinship found. Table S7. Detail on Pathogenic/Likely Pathogenic Germline Mutation. [file 12967_2023_4316_MOESM1_ESM.docx]

**Additional file**

**Table S1. The UKB data-field ID for each phenotype used in this study.**

**Table S2. Benign prostatic hyperplasia association results for all 27 reported genome-wide significant risk variants.**

**Table S3. Details on SNP information of PRACTICAL PRS.**

**Table S4. Details on SNP information of PHS.**

**Table S5. Demographic and clinical characteristics of British white males in UK Biobank.**

**Table S6. Associations between BPH/PV-related PRS and risk of prostate cancer-specific mortality in participants with no kinship found.**

**Table S7. Detail on Pathogenic/Likely Pathogenic Germline Mutation.**

**Table S1. The UKB data-field ID for each phenotype used in this study.**

| **Data-Field** | **Description** |
| --- | --- |
| 21022 | Age at recruitment |
| 31 | Sex |
| 22001 | Genetic sex |
| 21000 | Ethnic background |
| 191 | Date lost to follow-up |
| 22021 | Genetic kinship to other participants |
| 22027 | Outliers for heterozigosity or missing rate |
| 22019 | Sex chromosome aneuploidy |
| 22000 | Genotype measurement batch |
| 40006 | Type of cancer: ICD10 |
| 40005 | Date of cancer diagnosis |
| 41270 | Diagnoses - ICD10 |
| 132072 | Date N40 first reported (hyperplasia of prostate) |
| 40000 | Date of death |
| 40007 | Age at death |
| 40001 | Underlying (primary) cause of death: ICD10 |

**Table S2. Benign prostatic hyperplasia association results for all 27 reported genome-wide significant risk variants.**

| CHR | SNP | EA | OA |  | Caucasian-specific GWAS | | | | | | | | |
| --- | --- | --- | --- | --- | --- | --- | --- | --- | --- | --- | --- | --- | --- |
|  |  |  |  | Origin Dataset (Cases vs Controls) | | PMID | EAF | OR^1^ | 95%CI | P value | Meta-OR^2^ | 95%CI | P value^1^ |
| 2 | rs2556378 | T | G | Icelandic BPH/LUTs (9443/104000) | | 30410027 | 0.154 | 1.09 | 1.04-1.15 | 9.20E-04 | 1.12 | 1.08-1.15 | 3.40E-12 |
| 2 | rs10180282 | T | C | Icelandic BPH/LUTs (9443/104000) | | 30410027 | 0.456 | 1.07 | 1.03-1.11 | 8.70E-04 | 1.06 | 1.03-1.08 | 8.70E-07 |
| 5 | rs381949 | A | G | Icelandic BPH/LUTs (9443/104000) | | 30410027 | 0.415 | 0.90 | 0.86-0.93 | 7.80E-08 | 0.9 | 0.88-0.92 | 4.90E-19 |
| 5 | rs2853677 | G | A | Icelandic BPH/LUTs (9443/104000) | | 30410027 | 0.421 | 1.07 | 1.03-1.12 | 5.00E-04 | 1.09 | 1.06-1.11 | 1.70E-12 |
| 5 | rs10054105 | G | T | Icelandic BPH/LUTs (9443/104000) | | 30410027 | 0.213 | 0.91 | 0.87-0.96 | 9.20E-05 | 0.91 | 0.88-0.93 | 3.50E-12 |
| 5 | rs677394* | G | C | Icelandic BPH/LUTs (9443/104000) | | 30410027 | 0.123 | 0.92 | 0.87-0.97 | 2.90E-03 | 0.88 | 0.85-0.92 | 2.90E-11 |
| 6 | rs200476* | T | A | Icelandic BPH/LUTs (9443/104000) | | 30410027 | 0.162 | 0.85 | 0.80-0.90 | 7.90E-08 | 0.88 | 0.85-0.90 | 3.90E-17 |
| 10 | rs148678804 | A | G | Icelandic BPH/LUTs (9443/104000) | | 30410027 | 0.035 | 1.20 | 1.09-1.33 | 1.80E-04 | 1.27 | 1.19-1.35 | 3.00E-14 |
| 10 | rs7906649 | G | A | Icelandic BPH/LUTs (9443/104000) | | 30410027 | 0.286 | 1.06 | 1.02-1.11 | 6.00E-03 | 1.07 | 1.04-1.10 | 2.10E-07 |
| 10 | rs11199879 | C | T | Icelandic BPH/LUTs (9443/104000) | | 30410027 | 0.252 | 1.10 | 1.05-1.14 | 1.90E-05 | 1.14 | 1.11-1.17 | 5.70E-23 |
| 10 | rs4548546 | T | C | Icelandic BPH/LUTs (9443/104000) | | 30410027 | 0.31 | 1.08 | 1.04-1.13 | 2.90E-04 | 1.11 | 1.08-1.13 | 2.00E-16 |
| 10 | rs2981575 | G | A | Icelandic BPH/LUTs (9443/104000) | | 30410027 | 0.427 | 0.94 | 0.90-0.97 | 1.10E-03 | 0.94 | 0.92-0.96 | 6.00E-08 |
| 11 | rs72878024 | A | G | Icelandic BPH/LUTs (9443/104000) | | 30410027 | 0.08 | 0.82 | 0.77-0.88 | 7.40E-08 | 0.85 | 0.82-0.89 | 1.40E-12 |
| 12 | rs2555019 | T | C | Icelandic BPH/LUTs (9443/104000) | | 30410027 | 0.456 | 0.92 | 0.89-0.96 | 4.10E-05 | 0.93 | 0.91-0.95 | 2.40E-11 |
| 12 | rs8853 | C | T | Icelandic BPH/LUTs (9443/104000) | | 30410027 | 0.494 | 1.07 | 1.03-1.11 | 9.80E-04 | 1.07 | 1.05-1.10 | 1.40E-09 |
| 13 | rs1638703* | C | G | Icelandic BPH/LUTs (9443/104000) | | 30410027 | 0.256 | 1.09 | 1.04-1.14 | 8.70E-05 | 1.1 | 1.07-1.13 | 1.10E-13 |
| 13 | rs6561599* | C | G | Icelandic BPH/LUTs (9443/104000) | | 30410027 | 0.371 | 0.94 | 0.90-0.98 | 2.30E-03 | 0.94 | 0.92-0.96 | 1.80E-07 |
| 17 | rs11651052 | A | G | Icelandic BPH/LUTs (9443/104000) | | 30410027 | 0.47 | 0.95 | 0.91-0.98 | 5.50E-03 | 0.93 | 0.91-0.95 | 3.20E-10 |
| 18 | rs9958656 | T | C | Icelandic BPH/LUTs (9443/104000) | | 30410027 | 0.43 | 1.11 | 1.07-1.15 | 1.50E-07 | 1.11 | 1.08-1.13 | 4.30E-19 |
| 18 | rs17670370 | G | T | Icelandic BPH/LUTs (9443/104000) | | 30410027 | 0.262 | 1.05 | 1.01-1.09 | 2.70E-02 | 1.07 | 1.04-1.10 | 1.60E-07 |
| 19 | rs11084596 | C | T | Icelandic BPH/LUTs (9443/104000) | | 30410027 | 0.356 | 0.90 | 0.86-0.93 | 2.40E-07 | 0.88 | 0.86-0.90 | 2.10E-24 |
| 20 | rs200383755^#^ | C | G | Icelandic BPH/LUTs (9443/104000) | | 30410027 | 0.009 | 0.65 | 0.54-0.78 | 3.00E-06 | 0.67 | 0.59-0.77 | 3.20E-09 |
| 20 | rs6061244* | C | G | Icelandic BPH/LUTs (9443/104000) | | 30410027 | 0.386 | 0.96 | 0.92-1.00 | 4.60E-02 | 0.94 | 0.92-0.96 | 5.70E-08 |
| 2 | rs10192133 | C | T | eMERGE (1942/4730) | | 33219367 | 0.276 | 1.29 | 1.18-1.41 | 7.48E-09 | N/A | N/A | N/A |
| 11 | rs1237696 | T | C | eMERGE (1942/4730) | | 33219367 | 0.244 | 1.36 | 1.22-1.52 | 4.21E-08 | N/A | N/A | N/A |
| 15 | rs8027714 | A | G | eMERGE (1942/4730) | | 33219367 | 0.163 | 0.66 | 0.59-0.74 | 5.62E-12 | N/A | N/A | N/A |
| 22 | rs8136152 | G | A | eMERGE (1942/4730) | | 33219367 | 0.171 | 0.72 | 0.65-0.81 | 3.09E-09 | N/A | N/A | N/A |

Abbreviation: CHR, Chromosome; EA, Effective allele; OA, Other allele; BPH, Benign prostatic hyperplasia; LUTs, Lower urinary tract symptoms; EAF, Effective allele frequency; OR, Odds ratio.

1. Used in the weighted PRS calculation.
2. Combined results, which were validated in UK Biobank in the reported GWAS.

* SNPs not used for PRS calculation due to ambiguous variants.

# SNPs absent in UKB imputed genotyping data.

**Table S3. Details on SNP informations of PRACTICAL PRS.**

| **SNP** | **Chr** | **Position** | **A1** | **Beta** | **Inclusion** |
| --- | --- | --- | --- | --- | --- |
| rs56391074 | 1 | 88210715 | AT | 0.047 | No SNP |
| rs34579442 | 1 | 153899900 | C | 0.066 | No SNP |
| rs17599629 | 1 | 150658287 | G | 0.065 | Yes |
| rs1218582 | 1 | 154834183 | G | 0.046 | Yes |
| rs4245739 | 1 | 204518842 | A | 0.092 | Yes |
| rs62106670 | 2 | 8597123 | T | 0.052 | No SNP |
| rs74702681 | 2 | 66652885 | T | 0.159 | Yes |
| rs11691517 | 2 | 111893096 | T | 0.064 | Yes |
| rs34925593 | 2 | 174234547 | C | 0.047 | Yes |
| rs59308963 | 2 | 202123479 | T | 0.051 | No SNP |
| rs9287719 | 2 | 10710730 | C | 0.066 | Yes |
| rs9306895 | 2 | 20878153 | C | 0.077 | Yes |
| rs1465618 | 2 | 43553949 | T | 0.083 | Yes |
| rs721048 | 2 | 63131731 | A | 0.097 | Yes |
| rs10187424 | 2 | 85794297 | T | 0.074 | Yes |
| rs12621278 | 2 | 173311553 | A | 0.242 | Yes |
| rs2292884 | 2 | 238443226 | G | 0.061 | Yes |
| rs3771570 | 2 | 242382864 | T | 0.084 | Yes |
| rs1283104 | 3 | 106962521 | G | 0.047 | Yes |
| rs182314334 | 3 | 152004202 | T | 0.089 | Yes |
| rs142436749 | 3 | 169093100 | G | 0.221 | Yes |
| rs2660753 | 3 | 87110674 | T | 0.12 | Yes |
| rs7611694 | 3 | 113275624 | A | 0.083 | Yes |
| rs10934853 | 3 | 128038373 | A | 0.099 | Yes |
| rs6763931 | 3 | 141102833 | A | 0.043 | Yes |
| rs10936632 | 3 | 170130102 | A | 0.097 | Yes |
| rs10009409 | 4 | 73855253 | T | 0.056 | Yes |
| rs1894292 | 4 | 74349158 | G | 0.062 | Yes |
| rs12500426 | 4 | 95514609 | A | 0.069 | Yes |
| rs17021918 | 4 | 95562877 | C | 0.085 | Yes |
| rs7679673 | 4 | 106061534 | C | 0.12 | Yes |
| rs10793821 | 5 | 133836209 | T | 0.053 | Yes |
| rs76551843 | 5 | 169172133 | A | 0.271 | No SNP |
| rs4976790 | 5 | 177968915 | T | 0.074 | Yes |
| rs2242652 | 5 | 1280028 | G | 0.16 | Yes |
| rs12653946 | 5 | 1895829 | T | 0.079 | Yes |
| rs2121875 | 5 | 44365545 | C | 0.048 | Yes |
| rs12665339 | 6 | 30601232 | G | 0.062 | Yes |
| rs9296068 | 6 | 32988695 | T | 0.048 | Yes |
| rs9469899 | 6 | 34793124 | A | 0.048 | Yes |
| rs4711748 | 6 | 43694598 | T | 0.052 | Yes |
| rs4713266 | 6 | 11219030 | C | 0.051 | Yes |
| rs7767188 | 6 | 30073776 | A | 0.054 | Yes |
| rs3096702 | 6 | 32192331 | A | 0.056 | Yes |
| rs3129859 | 6 | 32400939 | G | 0.06 | Yes |
| rs1983891 | 6 | 41536427 | T | 0.082 | Yes |
| rs9443189 | 6 | 76495882 | A | 0.064 | Yes |
| rs2273669 | 6 | 109285189 | G | 0.069 | Yes |
| rs339331 | 6 | 117210052 | T | 0.084 | Yes |
| rs1933488 | 6 | 153441079 | A | 0.076 | Yes |
| rs9364554 | 6 | 160833664 | T | 0.104 | Yes |
| rs527510716 | 7 | 1944537 | C | 0.059 | No SNP |
| rs11452686 | 7 | 20414110 | T | 0.05 | No SNP |
| rs17621345 | 7 | 40875192 | A | 0.072 | Yes |
| rs12155172 | 7 | 20994491 | A | 0.093 | Yes |
| rs10486567 | 7 | 27976563 | G | 0.134 | Yes |
| rs56232506 | 7 | 47437244 | A | 0.054 | Yes |
| rs6465657 | 7 | 97816327 | C | 0.101 | Yes |
| rs2928679 | 8 | 23438975 | A | 0.053 | Yes |
| rs1512268 | 8 | 23526463 | T | 0.128 | Yes |
| rs11135910 | 8 | 25892142 | T | 0.078 | Yes |
| rs12543663 | 8 | 127924659 | C | 0.111 | Yes |
| rs10086908 | 8 | 128011937 | T | 0.126 | Yes |
| rs183373024 | 8 | 128104117 | G | 1.068 | No SNP |
| rs16901979 | 8 | 128124916 | A | 0.445 | Yes |
| rs620861 | 8 | 128335673 | G | 0.139 | Yes |
| rs6983267 | 8 | 128413305 | G | 0.2 | Yes |
| rs1447295 | 8 | 128485038 | A | 0.345 | Yes |
| rs1048169 | 9 | 19055965 | C | 0.061 | Yes |
| rs10122495 | 9 | 34049779 | T | 0.05 | Yes |
| rs1182 | 9 | 132576060 | A | 0.058 | Yes |
| rs17694493 | 9 | 22041998 | G | 0.073 | Yes |
| rs141536087 | 10 | 854691 | GCGCA | 0.081 | No SNP |
| rs1935581 | 10 | 90195149 | C | 0.048 | Yes |
| rs7094871 | 10 | 114712154 | G | 0.044 | Yes |
| rs76934034 | 10 | 46082985 | T | 0.115 | Yes |
| rs10993994 | 10 | 51549496 | T | 0.208 | Yes |
| rs3850699 | 10 | 104414221 | A | 0.07 | Yes |
| rs4962416 | 10 | 126696872 | C | 0.059 | Yes |
| rs1881502 | 11 | 1507512 | T | 0.058 | Yes |
| rs61890184 | 11 | 7547587 | A | 0.071 | Yes |
| rs547171081 | 11 | 47421962 | CGG | 0.047 | No SNP |
| rs2277283 | 11 | 61908440 | C | 0.056 | Yes |
| rs12785905 | 11 | 66951965 | C | 0.116 | Yes |
| rs11290954 | 11 | 76260543 | AC | 0.061 | No SNP |
| rs1800057 | 11 | 108143456 | G | 0.15 | Yes |
| rs138466039 | 11 | 125054793 | T | 0.281 | Yes |
| rs878987 | 11 | 134266372 | G | 0.064 | Yes |
| rs7127900 | 11 | 2233574 | A | 0.17 | Yes |
| rs7931342 | 11 | 68994497 | G | 0.157 | Yes |
| rs11568818 | 11 | 102401661 | T | 0.074 | Yes |
| rs11214775 | 11 | 113807181 | G | 0.071 | Yes |
| rs2066827 | 12 | 12871099 | T | 0.056 | Yes |
| rs10845938 | 12 | 14416918 | G | 0.057 | Yes |
| rs7968403 | 12 | 65012824 | T | 0.059 | Yes |
| rs5799921 | 12 | 90160530 | GA | 0.061 | No SNP |
| rs7295014 | 12 | 133067989 | G | 0.052 | Yes |
| rs80130819 | 12 | 48419618 | A | 0.096 | Yes |
| rs10875943 | 12 | 49676010 | C | 0.069 | Yes |
| rs902774 | 12 | 53273904 | A | 0.126 | Yes |
| rs1270884 | 12 | 114685571 | A | 0.07 | Yes |
| rs1004030 | 14 | 23305649 | T | 0.046 | Yes |
| rs11629412 | 14 | 37138294 | C | 0.057 | Yes |
| rs8008270 | 14 | 53372330 | C | 0.083 | Yes |
| rs7141529 | 14 | 69126744 | C | 0.051 | Yes |
| rs8014671 | 14 | 71092256 | G | 0.047 | Yes |
| rs4924487 | 15 | 40922915 | C | 0.062 | Yes |
| rs33984059 | 15 | 56385868 | A | 0.176 | Yes |
| rs112293876 | 15 | 66764641 | C | 0.057 | No SNP |
| rs11863709 | 16 | 57654576 | C | 0.148 | Yes |
| rs201158093 | 16 | 82178893 | TAA | 0.049 | No SNP |
| rs28441558 | 17 | 7803118 | C | 0.151 | Yes |
| rs142444269 | 17 | 30098749 | C | 0.067 | Yes |
| rs2680708 | 17 | 56456120 | G | 0.046 | Yes |
| rs684232 | 17 | 618965 | C | 0.083 | Yes |
| rs11649743 | 17 | 36074979 | G | 0.122 | Yes |
| rs4430796 | 17 | 36098040 | A | 0.197 | Yes |
| rs138213197 | 17 | 46805705 | T | 1.348 | No SNP |
| rs11650494 | 17 | 47345186 | A | 0.099 | Yes |
| rs1859962 | 17 | 69108753 | G | 0.161 | Yes |
| rs8093601 | 18 | 51772473 | C | 0.045 | Yes |
| rs28607662 | 18 | 53230859 | C | 0.075 | Yes |
| rs12956892 | 18 | 56746315 | T | 0.05 | Yes |
| rs533722308 | 18 | 60961193 | CT | 0.052 | No SNP |
| rs10460109 | 18 | 73036165 | T | 0.044 | Yes |
| rs7241993 | 18 | 76773973 | C | 0.076 | Yes |
| rs11666569 | 19 | 17214073 | C | 0.052 | Yes |
| rs118005503 | 19 | 32167803 | G | 0.09 | Yes |
| rs61088131 | 19 | 42700947 | T | 0.062 | Yes |
| rs8102476 | 19 | 38735613 | C | 0.09 | Yes |
| rs11672691 | 19 | 41985587 | G | 0.092 | Yes |
| rs2735839 | 19 | 51364623 | G | 0.167 | Yes |
| rs11480453 | 20 | 31347512 | C | 0.046 | No SNP |
| rs6091758 | 20 | 52455205 | G | 0.072 | Yes |
| rs12480328 | 20 | 49527922 | T | 0.107 | Yes |
| rs2427345 | 20 | 61015611 | C | 0.045 | Yes |
| rs6062509 | 20 | 62362563 | T | 0.078 | Yes |
| rs1041449 | 21 | 42901421 | G | 0.051 | Yes |
| rs9625483 | 22 | 28888939 | A | 0.134 | Yes |
| rs58133635 | 22 | 40471188 | T | 0.068 | Yes |
| rs5759167 | 22 | 43500212 | G | 0.142 | Yes |
| rs17321482 | 23 | 11482634 | C | 0.067 | Yes |
| rs2405942 | 23 | 9814135 | A | 0.049 | Yes |
| rs5945619 | 23 | 51241672 | C | 0.104 | Yes |
| rs2807031 | 23 | 52896949 | C | 0.058 | Yes |
| rs5919432 | 23 | 67021550 | T | 0.043 | Yes |
| rs6625711 | 23 | 70139850 | A | 0.008 | Yes |

**Table S4. Details on SNP informations of PHS.**

| **SNP** | **Chr** | **Position** | **A1** | **Beta** | **Inclusion** |
| --- | --- | --- | --- | --- | --- |
| rs6545977 | 2 | 63301164 | A | -0.069 | Yes |
| rs1010 | 2 | 85808982 | G | -0.049 | No matched allele |
| rs16860513 | 2 | 173342367 | T | -0.198 | Yes |
| rs75219487 | 3 | 87147922 | T | 0.123 | Yes |
| rs6788616 | 3 | 87205079 | G | 0.041 | Yes |
| rs4857841 | 3 | 128046643 | A | 0.031 | Yes |
| rs78416326 | 3 | 170074517 | G | -0.08 | Yes |
| rs6853490 | 4 | 95544718 | G | 0.053 | Yes |
| rs17021918 | 4 | 95562877 | A | -0.026 | No matched allele |
| rs7679673 | 4 | 106061534 | A | -0.071 | Yes |
| rs7725218 | 5 | 1282414 | A | -0.078 | Yes |
| rs2736108 | 5 | 1297488 | A | 0.056 | No matched allele |
| rs10866527 | 5 | 1891800 | A | 0.045 | No matched allele |
| rs3910736 | 6 | 153412476 | A | -0.073 | No matched allele |
| rs7769879 | 6 | 160865645 | G | 0.053 | Yes |
| rs6965016 | 7 | 97807882 | C | 0.049 | Yes |
| rs11782388 | 8 | 23525358 | G | 0.062 | No matched allele |
| rs9297746 | 8 | 127909361 | G | -0.061 | No matched allele |
| rs28556804 | 8 | 128014315 | G | -0.074 | Yes |
| rs77541621 | 8 | 128077146 | A | 0.2 | Yes |
| rs1016343 | 8 | 128093297 | A | 0.073 | No matched allele |
| rs60163266 | 8 | 128323157 | A | 0.073 | Yes |
| rs6983267 | 8 | 128413305 | A | -0.1 | No matched allele |
| rs7812894 | 8 | 128520479 | T | 0.084 | Yes |
| rs12549761 | 8 | 128540776 | G | -0.065 | Yes |
| rs10993994 | 10 | 51549496 | A | 0.106 | No matched allele |
| rs72853963 | 11 | 2224664 | A | 0.071 | Yes |
| rs12275055 | 11 | 68981359 | G | 0.083 | Yes |
| rs7929962 | 11 | 68985583 | G | -0.047 | No matched allele |
| rs11568818 | 11 | 102401661 | G | -0.047 | No matched allele |
| rs10875943 | 12 | 49676010 | G | 0.036 | No matched allele |
| rs55914512 | 12 | 53282274 | A | 0.05 | No matched allele |
| rs4643253 | 14 | 69106108 | G | -0.049 | No matched allele |
| rs684232 | 17 | 618965 | G | 0.042 | No matched allele |
| rs718961 | 17 | 36077099 | A | -0.074 | Yes |
| rs11651052 | 17 | 36102381 | A | -0.094 | Yes |
| rs117576373 | 17 | 46820676 | A | 0.138 | No matched allele |
| rs9889335 | 17 | 69115146 | A | 0.082 | No matched allele |
| rs11672691 | 19 | 41985587 | A | -0.062 | Yes |
| rs17632542 | 19 | 51361757 | G | -0.16 | No matched allele |
| rs35897249 | 20 | 62233638 | G | -0.049 | Yes |
| rs73179053 | 22 | 43501620 | G | -0.081 | No matched allele |
| rs747745 | 22 | 43503547 | G | -0.047 | No matched allele |
| rs4907775 | 23 | 51263200 | G | 0.056 | Yes |
| rs7888856 | 23 | 66751555 | G | -0.047 | Yes |
| rs11795627 | 23 | 69957441 | A | -0.04 | Yes |

**Table S5. Demographic and clinical characteristics of Caucasian population in UK Biobank.**

| **Characteristics** | **PCa Group** | **Non-PCa Group** | **Total** | **P value^1^** |
| --- | --- | --- | --- | --- |
| **Participants, No. (%)** | 14,549 (6.9) | 194,953 (93.1) | 209,502 |  |
| **Age at recruitment, mean (SD), yr** | 62.1 (5.5) | 56.6 (8.2) | 57.0 (8.1) | <0.001 |
| **BPH Diagnosis, No. (%)** |  |  |  | <0.001 |
| **Yes** | 2,808 (19.3) | 20,063 (10.3) | 22,871(10.9) |  |
| **No** | 11,741 (80.7) | 174,890 (89.7) | 186,631 (89.1) |  |
| **Family History of PCa, No. (%)** |  |  |  | <0.001 |
| **Yes** | 1,731 (11.9) | 16,426 (8.4) | 18,157 (8.1) |  |
| **No** | 12,201 (83.9) | 171,205 (87.8) | 183,406 (87.9) |  |
| **Missing** | 617 (4.2) | 7,322 (3.8) | 7,677 (4.0) |  |
| **Charlson comorbidity index scores, No. (%)** |  |  |  | <0.001 |
| **0** | 4,549 (31.3) | 137,185 (70.3) | 141,734 (67.6) |  |
| **1-2** | 6,018 (41.3) | 40,670 (20.9) | 46,688 (22.3) |  |
| **>2** | 3,982 (27.4) | 17,098 (8.8) | 21,080 (10.1) |  |
| **Genetic kinship** |  |  |  | 0.30 |
| **No kinship found** | 10,091 (69.4) | 135,777 (69.6) | 145,868 (69.6) |  |
| **At least one relative identified** | 4,450 (30.6) | 59,102 (30.3) | 63,552 (30.3) |  |
| **Ten or more third-degree relatives** | 8 (0.0) | 74 (0.1) | 82 (0.1) |  |
| **Lethal PCa^2^, No. (%)** | 959 (6.6) | 0 | 959 (0.5) |  |
| **No. PCa patients before recruitment, No. (%)** | 3715 (25.5) | 0 | 3715 (1.8) |  |

Abbreviation: PCa, Prostate cancer; BPH, Benign prostatic hyperplasia; SD, Standard deviation.

1. Chi-squared test or t test was used to compare PCa group and Non-PCa group.
2. Lethal PCa was defined as patients whose primary cause of death was PCa.

**Table S6. Associations between BPH/PV-related PRS and risk of prostate cancer-specific mortality in participants with no kinship found.**

| **PCa cohort** | **PCa** | **Lethal PCa** | **HR* (95%CI)** | ***P*** |
| --- | --- | --- | --- | --- |
| **All lethal PCa vs. others** | | | | |
| **Prevalence (n=145,868)** | 10,091 | 644 | 0.92 (0.85-0.99) | 0.03 |
| **Incident lethal PCa vs. other** | | | | |
| **Incidence (n=143,846)** | 8,069 | 464 | 0.91 (0.83-0.99) | 0.04 |
| **Lethal PCa vs. Non lethal PCa** | | | | |
| **Prognosis (n=10,091)** | 10,091 | 644 | 0.91 (0.84-0.99) | 0.02 |

* HR is calculated by cox model adjusted for age, charlson comorbidity index score, genotyping chip batches and 10 principal components.

**Table S7. Detail on Pathogenic/Likely Pathogenic Germline Mutations.**

| **Gene** | **Chr** | **Start** | **End** | **Ref** | **Alt** | **Mutation** | **Transcript** | **HGVS_C** | **HGVS_P** | **ACMG** |
| --- | --- | --- | --- | --- | --- | --- | --- | --- | --- | --- |
| BRCA2 | 13 | 32356472 | 32356472 | C | T | stopgain | NM_000059 | c.C7480T | p.R2494X | PVS1;PM2;PS1 |
| BRCA2 | 13 | 32340037 | 32340037 | C | G | stopgain | NM_000059 | c.C5682G | p.Y1894X | PVS1;PM2;PS1 |
| BRCA2 | 13 | 32340414 | 32340417 | AACA | - | frameshift deletion | NM_000059 | c.6059_6062del | p.E2020fs | PVS1;PM2;PS1 |
| BRCA2 | 13 | 32332867 | 32332868 | AG | - | frameshift deletion | NM_000059 | c.1389_1390del | p.T463fs | PVS1;PM2;PS1 |
| BRCA2 | 13 | 32337760 | 32337760 | C | A | stopgain | NM_000059 | c.C3405A | p.Y1135X | PVS1;PM2;PS1 |
| BRCA2 | 13 | 32326517 | 32326517 | - | AT | frameshift insertion | NM_000059 | c.535_536insAT | p.H179fs | PVS1;PM2;PS1 |
| BRCA2 | 13 | 32338828 | 32338831 | GAAA | - | frameshift deletion | NM_000059 | c.4473_4476del | p.L1491fs | PVS1;PM2;PS1 |
| BRCA2 | 13 | 32326615 | 32326615 | T | G | splicing | NM_000059 | c.631+2T>G | . | PVS1;PM2;PS1 |
| BRCA2 | 13 | 32336762 | 32336762 | - | A | stopgain | NM_000059 | c.2408dupA | p.Y803_E804delinsX | PVS1;PM2 |
| BRCA2 | 13 | 32338201 | 32338202 | TG | - | frameshift deletion | NM_000059 | c.3846_3847del | p.T1282fs | PVS1;PM2 |
| BRCA2 | 13 | 32339929 | 32339932 | AATT | - | frameshift deletion | NM_000059 | c.5574_5577del | p.T1858fs | PVS1;PM2;PS1 |
| BRCA2 | 13 | 32339229 | 32339230 | AA | - | frameshift deletion | NM_000059 | c.4874_4875del | p.E1625fs | PVS1;PM2;PS1 |
| BRCA2 | 13 | 32340630 | 32340631 | TT | - | frameshift deletion | NM_000059 | c.6275_6276del | p.L2092fs | PVS1;PM2;PS1 |
| BRCA2 | 13 | 32371001 | 32371002 | AG | - | frameshift deletion | NM_000059 | c.8533_8534del | p.R2845fs | PVS1;PM2;PS1 |
| BRCA2 | 13 | 32326615 | 32326615 | T | G | splicing | NM_000059 | c.631+2T>G | . | PVS1;PM2;PS1 |
| BRCA2 | 13 | 32339214 | 32339214 | T | G | stopgain | NM_000059 | c.T4859G | p.L1620X | PVS1;PM2;PS1 |
| BRCA2 | 13 | 32339658 | 32339659 | TT | - | frameshift deletion | NM_000059 | c.5303_5304del | p.L1768fs | PVS1;PM2;PS1 |
| BRCA2 | 13 | 32331031 | 32331031 | G | A | splicing | NM_000059 | c.793+1G>A | . | PVS1;PM2;PS1 |
| BRCA2 | 13 | 32331010 | 32331011 | AA | - | frameshift deletion | NM_000059 | c.773_774del | p.Q258fs | PVS1;PM2;PS1 |
| BRCA2 | 13 | 32394734 | 32394734 | T | G | nonsynonymous SNV | NM_000059 | c.T9302G | p.L3101R | PM2;PS1 |
| BRCA2 | 13 | 32363390 | 32363390 | G | T | nonsynonymous SNV | NM_000059 | c.G8188T | p.A2730S | PM2;PM5 |
| BRCA2 | 13 | 32337161 | 32337164 | AAAC | - | frameshift deletion | NM_000059 | c.2806_2809del | p.K936fs | PVS1;PM2;PS1 |
| BRCA2 | 13 | 32340037 | 32340037 | C | G | stopgain | NM_000059 | c.C5682G | p.Y1894X | PVS1;PM2;PS1 |
| BRCA2 | 13 | 32340073 | 32340074 | CT | - | frameshift deletion | NM_000059 | c.5718_5719del | p.N1906fs | PVS1;PM2;PS1 |
| BRCA2 | 13 | 32330987 | 32330990 | GACA | - | frameshift deletion | NM_000059 | c.750_753del | p.V250fs | PVS1;PM2;PS1 |
| BRCA2 | 13 | 32339421 | 32339421 | - | A | frameshift insertion | NM_000059 | c.5067dupA | p.A1689fs | PVS1;PM2;PS1 |
| BRCA2 | 13 | 32336764 | 32336764 | T | G | stopgain | NM_000059 | c.T2409G | p.Y803X | PVS1;PM2;PS1 |
| BRCA2 | 13 | 32326613 | 32326613 | G | C | nonsynonymous SNV | NM_000059 | c.G631C | p.V211L | PM2;PS1 |
| BRCA2 | 13 | 32338765 | 32338768 | AAGA | - | frameshift deletion | NM_000059 | c.4410_4413del | p.I1470fs | PVS1;PM2;PS1 |
| BRCA2 | 13 | 32338140 | 32338140 | C | A | stopgain | NM_000059 | c.C3785A | p.S1262X | PVS1;PM2 |
| BRCA2 | 13 | 32357914 | 32357916 | AAG | - | nonframeshift deletion | NM_000059 | c.7790_7792del | p.2597_2598del | PM2;PS1 |
| BRCA2 | 13 | 32332375 | 32332375 | - | CA | frameshift insertion | NM_000059 | c.897_898insCA | p.V299fs | PVS1;PM2 |
| BRCA2 | 13 | 32346867 | 32346867 | T | - | frameshift deletion | NM_000059 | c.6978delT | p.S2326fs | PVS1;PM2;PS1 |
| BRCA2 | 13 | 32326497 | 32326497 | A | G | splicing | NM_000059 | c.517-2A>G | . | PVS1;PM2;PS1 |
| BRCA2 | 13 | 32363535 | 32363535 | T | A | splicing | NM_000059 | c.8331+2T>A | . | PVS1;PM2;PS1 |
| BRCA1 | 17 | 43093056 | 43093056 | G | - | frameshift deletion | NM_007294 | c.2475delC | p.D825fs | PVS1;PM2;PS1 |
| BRCA1 | 17 | 43092526 | 43092526 | T | - | frameshift deletion | NM_007294 | c.3005delA | p.N1002fs | PVS1;PM2;PS1 |
| BRCA1 | 17 | 43095924 | 43095924 | T | G | splicing | NM_007294 | c.594-2A>C | . | PVS1;PM2 |
| BRCA1 | 17 | 43104867 | 43104867 | C | T | splicing | NM_007294 | c.301+1G>A | . | PVS1;PM2;PS1 |
| BRCA1 | 17 | 43045767 | 43045767 | G | A | stopgain | NM_007294 | c.C5503T | p.R1835X | PVS1;PM2;PS1 |
| BRCA1 | 17 | 43124028 | 43124029 | CT | - | frameshift deletion | NM_007294 | c.68_69del | p.E23fs | PVS1;PM2;PS1 |
| BRCA1 | 17 | 43091924 | 43091924 | G | A | stopgain | NM_007294 | c.C3607T | p.R1203X | PVS1;PM2;PS1 |
| BRCA1 | 17 | 43091772 | 43091775 | AGAC | - | frameshift deletion | NM_007294 | c.3756_3759del | p.L1252fs | PVS1;PM2;PS1 |
| ATM | 11 | 108284281 | 108284281 | G | - | frameshift deletion | NM_000051 | c.3801delG | p.E1267fs | PVS1;PM2;PS1 |
| ATM | 11 | 108335959 | 108335959 | A | T | stopgain | NM_000051 | c.A8266T | p.K2756X | PVS1;PM2;PS1 |
| ATM | 11 | 108256340 | 108256340 | G | A | synonymous SNV | NM_000051 | c.G2250A | p.K750K | PM2;PS1 |
| ATM | 11 | 108301698 | 108301698 | C | T | nonsynonymous SNV | NM_000051 | c.C5228T | p.T1743I | PM2;PS1 |
| ATM | 11 | 108331885 | 108331893 | TCTAGAATT | - | nonframeshift deletion | NM_000051 | c.7636_7644del | p.2546_2548del | PM2;PS1 |
| ATM | 11 | 108354875 | 108354875 | G | A | splicing | NM_000051 | c.8850+1G>A | . | PVS1;PM2;PS1 |
| ATM | 11 | 108307928 | 108307928 | - | A | frameshift insertion | NM_000051 | c.5707dupA | p.D1902fs | PVS1;PM2;PS1 |
| ATM | 11 | 108279555 | 108279555 | C | T | stopgain | NM_000051 | c.C3349T | p.Q1117X | PVS1;PM2;PS1 |
| ATM | 11 | 108281168 | 108281168 | G | C | nonsynonymous SNV | NM_000051 | c.G3576C | p.K1192N | PM2;PS1 |
| ATM | 11 | 108289759 | 108289759 | T | C | nonsynonymous SNV | NM_000051 | c.T4394C | p.L1465P, | PM2;PS1 |
| ATM | 11 | 108272812 | 108272812 | - | TG | frameshift insertion | NM_000051 | c.3244_3245insTG | p.H1082fs | PVS1;PM2 |
| ATM | 11 | 108365476 | 108365476 | C | T | stopgain | NM_000051 | c.C9139T | p.R3047X | PVS1;PM2;PS1 |
| ATM | 11 | 108227626 | 108227626 | T | C | nonsynonymous SNV | NM_000051 | c.T2C | p.M1T | PM2;PS1 |
| ATM | 11 | 108317463 | 108317463 | G | T | stopgain | NM_000051 | c.G6289T | p.E2097X | PVS1;PM2;PS1 |
| ATM | 11 | 108316114 | 108316114 | G | A | splicing | NM_000051 | c.6198+1G>A | . | PVS1;PM2;PS1 |
| ATM | 11 | 108317463 | 108317463 | G | T | stopgain | NM_000051 | c.G6289T | p.E2097X | PVS1;PM2;PS1 |
| ATM | 11 | 108329202 | 108329202 | T | G | nonsynonymous SNV | NM_000051 | c.T7271G | p.V2424G | PM2;PS1 |
| ATM | 11 | 108304801 | 108304801 | C | T | stopgain | NM_000051 | c.C5623T | p.R1875X, | PVS1;PM2;PS1 |
| ATM | 11 | 108335105 | 108335105 | T | C | nonsynonymous SNV | NM_000051 | c.T8147C | p.V2716A | PM2;PS1 |
| ATM | 11 | 108302895 | 108302895 | G | - | frameshift deletion | NM_000051 | c.5362delG | p.G1788fs | PVS1;PM2 |
| ATM | 11 | 108229208 | 108229209 | AG | - | frameshift deletion | NM_000051 | c.216_217del | p.T72fs | PVS1;PM2 |
| ATM | 11 | 108332808 | 108332808 | - | GA | frameshift insertion | NM_000051 | c.7835_7836insGA | p.R2612fs | PVS1;PM2;PS1 |
| ATM | 11 | 108353881 | 108353881 | G | A | splicing | NM_000051 | c.8786+1G>A | . | PVS1;PM2;PS1 |
| ATM | 11 | 108227691 | 108227691 | C | T | stopgain | NM_000051 | c.C67T | p.R23X | PVS1;PM2;PS1 |
| ATM | 11 | 108301706 | 108301706 | G | - | frameshift deletion | NM_000051 | c.5236delG | p.G1746fs | PVS1;PM2 |
| ATM | 11 | 108345818 | 108345818 | C | T | nonsynonymous SNV | NM_000051 | c.C8494T | p.R2832C | PM2;PS1 |
| ATM | 11 | 108343372 | 108343375 | GTGA | - | splicing | . | . | . | PVS1;PM2;PS1 |
| ATM | 11 | 108227806 | 108227806 | C | T | stopgain | NM_000051 | c.C103T | p.R35X | PVS1;PM2;PS1 |
| ATM | 11 | 108235784 | 108235787 | TTCT | - | frameshift deletion | NM_000051 | c.446_449del | p.I149fs | PVS1;PM2;PS1 |
| ATM | 11 | 108302877 | 108302877 | A | - | frameshift deletion | NM_000051 | c.5344delA | p.K1782fs | PVS1;PM2 |
| ATM | 11 | 108229241 | 108229247 | AGCCTCA | - | frameshift deletion | NM_000051 | c.249_255del | p.S83fs, | PVS1;PM2 |
| ATM | 11 | 108331878 | 108331878 | G | A | splicing | NM_000051 | c.7630-1G>A | . | PVS1;PM2 |
| ATM | 11 | 108267183 | 108267183 | A | - | frameshift deletion | NM_000051 | c.2479delA | p.K827fs | PVS1;PM2;PS1 |
| HOXB13 | 17 | 48726830 | 48726830 | - | TC | frameshift insertion | NM_006361 | c.814_815insGA | p.K272fs | PVS1;PM2 |
| HOXB13 | 17 | 48728343 | 48728343 | C | T | nonsynonymous SNV | NM_006361 | c.G251A | p.G84E | PM2;PS1 |
| HOXB13 | 17 | 48728528 | 48728528 | T | - | frameshift deletion | NM_006361 | c.66delA | p.G22fs | PVS1;PM2 |
| CHEK2 | 22 | 28725099 | 28725099 | A | G | nonsynonymous SNV | NM_007194 | c.T470C | p.I157T | PM2;PS1 |
| CHEK2 | 22 | 28695869 | 28695869 | G | - | frameshift deletion | NM_007194 | c.1100delC | p.T367fs | PVS1;PM2;PS1 |
| CHEK2 | 22 | 28725084 | 28725086 | TCT | - | nonframeshift deletion | NM_007194 | c.483_485del | p.161_162del | PM2;PS1 |
| CHEK2 | 22 | 28695239 | 28695239 | A | - | frameshift deletion | NM_007194 | c.1263delT | p.L421fs | PVS1;PM2;PS1 |
| CHEK2 | 22 | 28694066 | 28694066 | G | A | nonsynonymous SNV | NM_007194 | c.C1427T | p.T476M | PM2;PS1 |
| CHEK2 | 22 | 28710005 | 28710005 | - | A | splicing | NM_007194 | c.846+1->T | . | PVS1;PM2 |
| CHEK2 | 22 | 28725278 | 28725278 | G | A | stopgain | NM_007194 | c.C409T | p.R137X | PVS1;PM2;PS1 |
| CHEK2 | 22 | 28725338 | 28725338 | T | C | nonsynonymous SNV | NM_007194 | c.A349G | p.R117G | PM2;PS1 |
| CHEK2 | 22 | 28734532 | 28734532 | C | T | nonsynonymous SNV | NM_007194 | c.G190A | p.E64K | PM2;PS1 |
| CHEK2 | 22 | 28725040 | 28725040 | T | A | stopgain | NM_007194 | c.A529T | p.K177X | PVS1;PM2;PS1 |
| CHEK2 | 22 | 28719423 | 28719423 | C | - | frameshift deletion | NM_007194 | c.655delG | p.E219fs | PVS1;PM2;PS1 |
| CHEK2 | 22 | 28734445 | 28734445 | A | - | frameshift deletion | NM_007194 | c.277delT | p.W93fs | PVS1;PM2;PS1 |
| CHEK2 | 22 | 28734439 | 28734439 | G | A | stopgain | NM_007194 | c.C283T | p.R95X | PVS1;PM2;PS1 |
| CHEK2 | 22 | 28689217 | 28689217 | T | C | splicing | NM_007194 | c.1462-2A>G | . | PVS1;PM2;PS1 |
| CHEK2 | 22 | 28725242 | 28725242 | C | T | splicing | NM_007194 | c.444+1G>A | . | PVS1;PM2;PS1 |
| PALB2 | 16 | 23630206 | 23630206 | C | - | frameshift deletion | NM_024675 | c.1948delG | p.E650fs | PVS1;PM2 |
| PALB2 | 16 | 23635000 | 23635000 | T | - | frameshift deletion | NM_024675 | c.1546delA | p.R516fs | PVS1;PM2;PS1 |
| PALB2 | 16 | 23614089 | 23614089 | T | - | frameshift deletion | NM_024675 | c.3116delA | p.N1039fs | PVS1;PM2;PS1 |
| PALB2 | 16 | 23621468 | 23621471 | TTTC | - | frameshift deletion | NM_024675 | c.3004_3007del | p.E1002fs | PVS1;PM2;PS1 |
| PALB2 | 16 | 23621362 | 23621362 | C | T | stopgain | NM_024675 | c.G3113A | p.W1038X | PVS1;PM2;PS1 |
| PALB2 | 16 | 23641155 | 23641155 | C | T | nonsynonymous SNV | NM_024675 | c.G3A | p.M1I, | PM2;PS1 |
| PALB2 | 16 | 23636058 | 23636059 | AC | - | frameshift deletion | NM_024675 | c.487_488del | p.V163fs | PVS1;PM2;PS1 |
| MLH1 | 3 | 37048994 | 37048994 | G | T | stopgain | NM_000249 | c.G2080T | p.E694X | PVS1;PM2 |
| MSH6 | 2 | 47806653 | 47806656 | TAAC | - | splicing | . | . | . | PVS1;PM2 |
| MSH6 | 2 | 47800645 | 47800645 | A | T | stopgain | NM_000179 | c.A2662T | p.K888X | PVS1;PM2 |
| MSH6 | 2 | 47806805 | 47806805 | C | G | stopgain | NM_000179 | c.C4028G | p.S1343X | PVS1;PM2 |
| MSH6 | 2 | 47803500 | 47803500 | - | C | frameshift insertion | NM_000179 | c.3254dupC | p.T1085fs | PVS1;PM2;PS1 |
| MSH6 | 2 | 47800056 | 47800056 | - | A | frameshift insertion | NM_000179 | c.2074dupA | p.L691fs | PVS1;PM2;PS1 |
| MSH6 | 7 | 5997387 | 5997387 | - | CTTCACACAC | frameshift insertion | NM_000535 | c.741_742insGTGTGTGAAG | p.S248fs | PVS1;PM2 |
| MSH6 | 7 | 6005918 | 6005918 | C | A | nonsynonymous SNV | NM_000535 | c.G137T | p.S46I | PM2;PS1 |
| MSH6 | 7 | 5997424 | 5997424 | - | T | splicing | NM_000535 | c.706-1->A | . | PVS1;PM2 |
| MSH6 | 7 | 6002590 | 6002590 | G | A | stopgain | NM_000535 | c.C400T | p.R134X | PVS1;PM2;PS1 |
